# Evaluating the Body Roundness Index as a Novel Digital Biomarker for Psoriasis Risk Prediction: Cross-Sectional Study

**DOI:** 10.2196/75727

**Published:** 2025-12-23

**Authors:** Pengfei Wen, Xiaoyan Wang, Xiaoxue Zhuo, Siliang Xue

**Affiliations:** 1 Department of Dermatology, West China Hospital, Sichuan University, No. 37 Guoxue Alley, Wuhou District, Chengdu, 610041, China, 86 13982262622; 2 Department of Clinical Nutrition, The First Affiliated Hospital of Chengdu Medical College, Chengdu, China

**Keywords:** psoriasis, body roundness index, obesity, digital biomarker, risk prediction

## Abstract

**Background:**

Psoriasis is a chronic inflammatory skin disorder that has been increasingly linked to metabolic imbalances, particularly obesity. Conventional anthropometric indicators such as BMI and waist circumference (WC) may not sufficiently capture body fat distribution or reflect metabolic risk. The body roundness index (BRI), which integrates both height and waist measurements, has emerged as a potentially superior metric, though its relevance to psoriasis risk remains underexplored.

**Objective:**

This study aimed to investigate the use of BRI as a digital biomarker for assessing psoriasis risk and to compare its predictive strength against BMI and WC across various demographic and metabolic subgroups using data from a nationally representative sample.

**Methods:**

A cross-sectional analysis was conducted using data from 13,798 adults aged 20 to 59 years who participated in the National Health and Nutrition Examination Survey between 2003 and 2006 as well as between 2009 and 2014. Psoriasis status was self-reported. Anthropometric measures (BRI, BMI, and WC) were calculated from standardized physical assessments. Weighted multivariable logistic regression models and restricted cubic spline analyses were used to examine associations while adjusting for demographic, metabolic, and lifestyle variables. A nomogram was constructed to quantify the relative predictive contributions of each metric.

**Results:**

BRI exhibited a strong linear association with psoriasis risk (odds ratio [OR] 1.11 per unit increase, 95% CI 1.05‐1.17; *P*<.001), outperforming BMI (OR 1.03) and WC (OR 1.01). Tertile analysis revealed a 1.73-fold increased risk of psoriasis in the highest BRI group (*P*=.003). Subgroup analyses confirmed consistent associations across age, sex, race or ethnicity, and metabolic status (*P* for interaction >.05). The nomogram highlighted BRI as the most influential predictor, indicated by its broad scoring range.

**Conclusions:**

BRI shows stronger and more consistent associations with psoriasis risk than BMI or WC, supporting its potential role as a digital biomarker for early risk stratification. Incorporating BRI into clinical decision-making tools may enhance personalized approaches to psoriasis prevention and management.

## Introduction

Psoriasis is a widespread, long-term inflammatory skin condition affecting approximately 2% to 3% of the global population [1,2]. Clinically, it is characterized by erythematous plaques, scaling, epidermal hyperplasia, and other symptoms, typically affecting the scalp, elbows, knees, and lower back [3,4]. The disease often follows a prolonged, relapsing course with considerable interindividual variation. Beyond its dermatologic manifestations, psoriasis is increasingly recognized as a systemic condition associated with several metabolic comorbidities, including obesity, diabetes, and cardiovascular disease (CVD) [1,5-7]. Chronic systemic inflammation may be a key mechanism linking psoriasis to metabolic disorders. The inflammatory milieu in psoriasis can disrupt insulin sensitivity and lipid metabolism, thereby promoting the development of metabolic diseases [2,8,9]. Understanding the interplay between psoriasis, obesity, and metabolic syndrome is thus crucial for improving early diagnosis and developing integrated intervention strategies.

Recent evidence underscores the strong association between psoriasis and metabolic diseases [10,11], particularly obesity. Obesity is considered a significant risk factor for psoriasis onset [12,13] and may also worsen disease severity [14]. In individuals with obesity, adipose tissue functions as an active endocrine organ, releasing proinflammatory cytokines, which induce chronic low-grade systemic inflammation [13,15,16]. These cytokines can trigger or exacerbate psoriatic inflammation [12,17]. Moreover, individuals with both obesity and metabolic syndrome tend to have a longer disease duration and more refractory clinical course, highlighting the potential value of weight management in psoriasis care [10,18].

Although BMI and waist circumference (WC) are commonly applied in clinical practice to assess obesity, they have notable limitations. BMI estimates general obesity based solely on weight-to-height ratio and cannot distinguish between fat and lean mass or identify fat distribution patterns [19]. Consequently, its application may result in inaccurate risk assessment in patients with psoriasis [20]. WC, while reflective of abdominal fat, lacks the precision to capture total or visceral fat distribution [21]. The body roundness index (BRI) is a recently developed anthropometric measure that combines WC and height, offering a potentially valuable alternative to traditional obesity metrics [22]. BRI more accurately estimates visceral and abdominal fat distribution and has demonstrated stronger associations with metabolic syndrome and obesity-related disorders [23,24]. Its potential application in predicting risk of psoriasis, however, remains underexplored.

While previous research has documented a linkage between BRI and risk of psoriasis [25], its comparative predictive performance against traditional measures such as BMI and WC across different population subgroups requires further validation. This study probes into the predictive use of BRI for psoriasis and to determine whether it offers superior risk stratification in comparison with conventional anthropometric indices. By leveraging large-scale national health data, we seek to support the development of more individualized screening and prevention strategies for psoriasis, thereby advancing both theoretical understanding and clinical management.

## Methods

### Data Source

This study is based on data from the National Health and Nutrition Examination Survey (NHANES) [26], conducted by the National Center for Health Statistics of the Centers for Disease Control and Prevention. NHANES is a nationally representative, ongoing cross-sectional survey aimed at evaluating the nutritional and health conditions of the US civilian population not living in institutions. It integrates in-home interviews with standardized health assessments conducted in mobile examination centers (MECs), offering comprehensive demographic, clinical, and laboratory data.

We analyzed data from the 2003 to 2006 and 2009 to 2014 NHANES cycles. The 2003‐2006 datasets included self-reported psoriasis information for adults aged 20 to 59 years, while the 2009‐2014 cycles provided relevant health and anthropometric data for participants aged 16 to 80 years. For consistency and comparability, our analysis focused on adults aged 20 to 59 years across all cycles.

### Study Population

This cross-sectional analysis included data from the 5 NHANES cycles: 2003‐2004, 2005‐2006, 2009‐2010, 2011‐2012, and 2013‐2014. Participants were included if they were aged 20 to 59 years and had complete data on psoriasis status and relevant anthropometric and clinical variables. The exclusion criteria were as follows: (1) missing data on psoriasis diagnosis; (2) missing height, WC, or weight data, which prevented BRI calculation; (3) missing key demographic or clinical variables, including poverty-income ratio (PIR), education, marital status, smoking status, alcohol consumption, BMI, CVD, diabetes, or metabolic syndrome.

### Definition and Measurement of Psoriasis and BRI

Psoriasis status was determined based on participants’ responses in the “Medical Conditions” section of the NHANES questionnaire. Respondents were considered to have psoriasis if they answered “yes” to the question: “Has a healthcare provider ever told you that you have psoriasis?” The validity of self-reported psoriasis has been previously established [27,28].

WC and height were collected by trained personnel at MECs following established measurement procedures. Participants were examined while barefoot and dressed in light clothing. Height was recorded at the anatomical point between the lower margin of the last palpable rib and the top of the iliac crest. The BRI was calculated as: BRI=364.2‐365.5×(1 - [WC(m)/2π]^2^/[0.5× height(m)]^2^)^½^ [29]. BRI was then grouped into tertiles based on its distribution in the sample: tertile 1 (<3.87, corresponding to < 33rd percentile), tertile 2 (≥3.87 to <5.61, corresponding to the 33rd-67th percentiles), and tertile 3 (≥5.61, corresponding to ≥ 67th percentile; Figure S1 in Multimedia Appendix 1). This standardized approach to psoriasis classification and BRI calculation ensures high reproducibility and provides a robust framework for subsequent analyses.

### Covariate Data Source

Based on prior literature, the following covariates were included in this study: age, sex, race or ethnicity, education level, marital status, PIR, smoking status, alcohol consumption, BMI, body fat percentage (BFP), CVD, metabolic syndrome, and diabetes.

#### Demographic Variables

Race or ethnicity was self-reported and classified into 4 categories: Mexican American, non-Hispanic Black, non-Hispanic White, and other Hispanic and other races. Marital status was classified as married, unmarried, cohabiting, or other [30]. Education level was categorized as: (1) high school or less, (2) some college education, and (3) college graduate or above. Age was treated as a continuous variable in the main analysis and dichotomized into 2 age brackets (20‐39 y and 40‐59 y) for subgroup analysis to explore age-related heterogeneity.

#### Lifestyle Factors

Smoking status was categorized into 3 groups: never smokers (individuals who had smoked fewer than 100 cigarettes in their lifetime), former smokers (those who had smoked at least 100 cigarettes but were not currently smoking), and current smokers (participants who had smoked over 100 cigarettes and continued smoking at the time of the survey) [30]. Alcohol consumption was stratified into 5 levels based on self-reported consumption over the previous year: never (lifetime consumption of fewer than 12 occasions), former (no alcohol intake in the past year but at least 12 past occasions), light (women drinking ≤1 time per day and men ≤2 times per day in the past year), moderate (women drinking ≤2 times per day and men ≤3 times per day), and heavy (women drinking ≥3 times per day and men ≥4 times per day) [31].

#### Anthropometric and Clinical Measures

BMI was determined as weight in kilograms divided by height in meters squared (kg m^–2^) [32]. BFP was tested using bioelectrical impedance analysis, conducted at the NHANES MEC using standardized equipment. BFP reflects the proportion of an individual’s body weight that is composed of fat and is calculated based on parameters such as height, weight, age, and sex, following the algorithm outlined in the official NHANES technical manual [32]. For statistical analysis, BFP was analyzed both as a continuous measure and by tertiles, aligning with the BRI grouping structure to explore its association with psoriasis risk across fat distribution levels.

#### Clinical Conditions

Diabetes was determined based on any of the following criteria: receipt of antidiabetic treatment, HbA_1c_≥6.5%, use of antidiabetic medications, oral glucose tolerance test results ≥11.1 mmol L^–1^, fasting blood glucose ≥ 7.0 mmol L^–1^, or random blood glucose ≥ 11.1 mmol L^–1^ [33, 34]. CVD was identified based on self-reported medical history of coronary artery disease, myocardial infarction, heart failure, stroke, or angina [35,36]. Metabolic syndrome was defined according to the Adult Treatment Panel III (2005) and International Diabetes Federation (2009) criteria [37,38].

### Statistical Analysis

This study used the fasting subsample from the NHANES dataset to ensure the reliability of metabolic indicator measurements. The fasting subsample includes participants who underwent blood sampling under standardized fasting conditions. According to NHANES analytic guidelines, the appropriate sampling weight for combined 10-year analyses was calculated using the formula: 10-year MEC weight=2-year MEC weight/5 [28].

We used weighted multivariable logistic regression models to estimate odds ratios (ORs) and 95% CIs, evaluating the association between the BRI and the risk of psoriasis. Three sequential models were sequentially applied: model 1 (unadjusted), model 2 (adjusted for demographic variables including race or ethnicity, age, sex, education level, PIR, and marital status), model 3 (further adjusted for smoking status, alcohol consumption, metabolic syndrome, diabetes, CVD, and NHANES survey cycle, as differences in sampling strategy, measurement methods, or public health trends across survey cycles could introduce systematic bias and act as potential confounders). Trend tests were performed to assess the robustness of its association with psoriasis. In Model 3, we also conducted a weighted restricted cubic spline (RCS) analysis to test the nonlinear relationship between BRI and psoriasis.

To comprehensively assess body size indicators in relation to psoriasis, we performed subgroup analyses and interaction tests to evaluate the role of BRI across different populations (by age, sex, race or ethnicity, CVD, metabolic syndrome, and diabetes), and further conducted logistic regression analyses on WC and BMI to examine their independent effects.

Descriptive statistics were computed by applying NHANES sampling weights. For continuous variables, we reported weighted averages along with their corresponding SDs. Categorical variables were described using unweighted frequencies and weighted proportions. Group comparisons were made using survey-weighted 2-tailed *t* tests for continuous measures and the Rao-Scott adjusted chi-square tests for categorical data. To evaluate the relationship between BRI and psoriasis, we fitted weighted multivariable logistic regression models, treating BRI both as a continuous variable and as categorical tertiles.

All statistical procedures were executed using R software (version 4.4.1; R Foundation for Statistical Computing), supplemented with Free Statistics software (version 1.9.2). NHANES-recommended survey weights were incorporated throughout to reflect the survey’s complex multistage design and to yield estimates generalizable to the US civilian population. A 2-sided *P*-value <.05 was deemed statistically significant.

### Ethical Considerations

This study followed the STROBE (Strengthening the Reporting of Observational Studies in Epidemiology) guidelines [39] to maintain high standards of methodological clarity and transparency. The NHANES study protocol received approval from the National Center for Health Statistics institutional review board, and all participants provided informed written consent. Since the dataset is anonymized and publicly accessible, no additional ethical review was necessary for this secondary analysis.

## Results

### Baseline Characteristics

**Figure 1.**
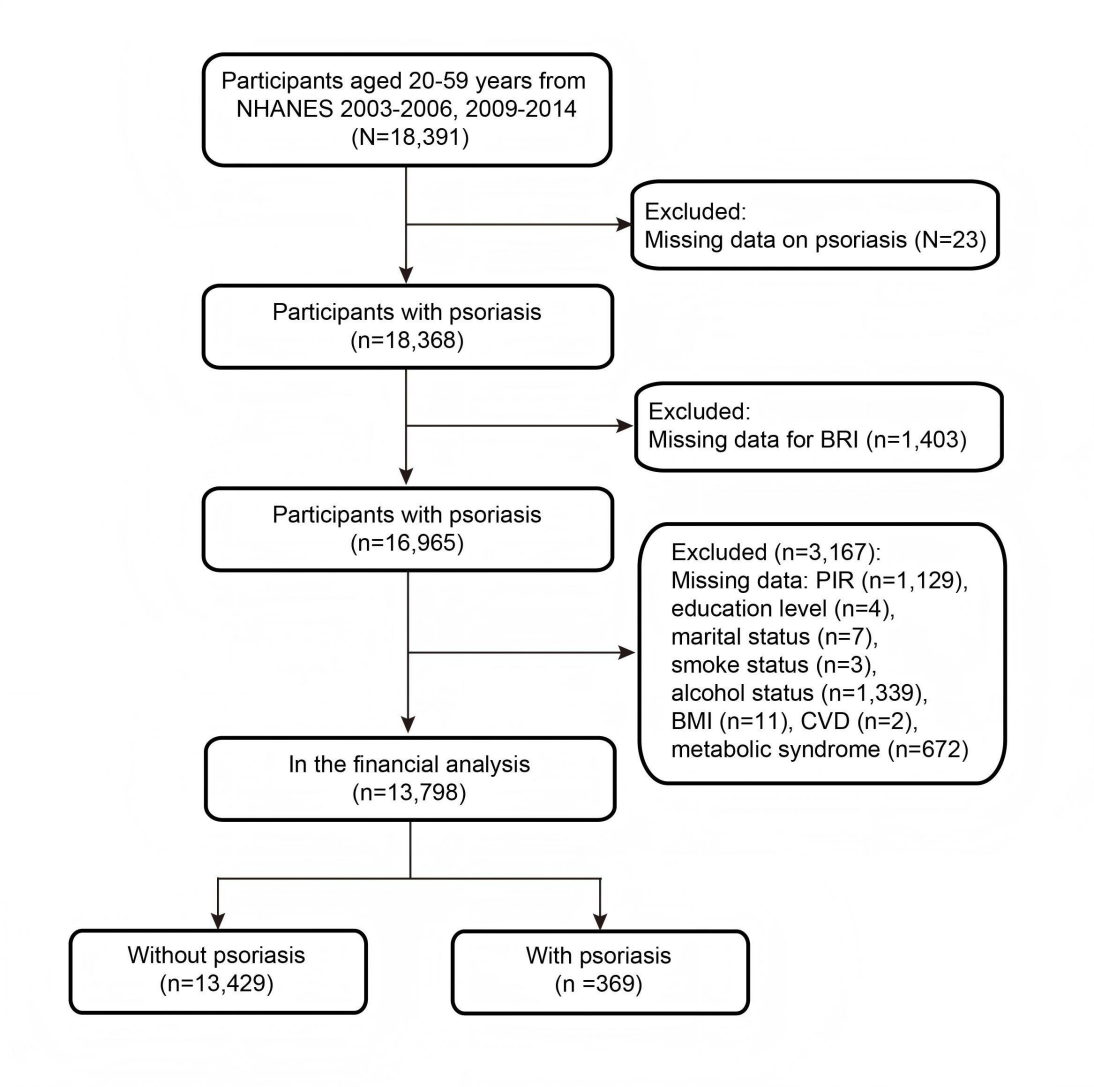
Flowchart of inclusion and exclusion criteria for patients with psoriasis. BRI: body roundness index; CVD: cardiovascular disease; NHANES: National Health and Nutrition Examination Survey.

The final analytic sample is detailed in the study flowchart (Figure 1). A total of 13,798 participants aged 20 to 59 years from the NHANES dataset were included in this study. Among them, 369 (3%) self-reported a diagnosis of psoriasis. The mean age of participants was 39.62 (SD 11.43) years. A total of 7037 (51%) participants were male and 6761 (49%) were female (Table 1). Participants were stratified into tertiles based on BRI, with tertile 1 (<3.87, n=4599), tertile 2 (3.87‐5.61, n=4599), and tertile 3 (≥5.61, n=4600). Significant differences in demographic, socioeconomic, and health-related characteristics were observed across tertiles.

In terms of demographic characteristics, individuals in tertile 3 (highest BRI ≥5.61) were older (mean 42.51, SD 10.79 y versus mean 35.57, SD 11.38 y in tertile 1). Female participants accounted for 2682 of 4600 (55.4%) participants in tertile 3, compared with 2104 of 4599 (49.4%) in tertile 1. In terms of race or ethnicity, non-Hispanic Whites were most common in tertile 3, with 1942 of 4600 (64.3%) participants, while Mexican Americans were least common, with 908 of 4600 (11.3%) participants. Regarding socioeconomic status, participants in tertile 3 had the lowest PIR and the highest proportion of individuals with low education levels, with 2307 of 4600 (44.1%) having only high school or less education. The proportion of married participants was also highest in tertile 3, with 2383 (56.8%).

**Table 1.** Weighted characteristics of participants in the National Health and Nutrition Examination Survey (NHANES) 2003‐2006 and 2009‐2014 cycles (N=13,798).

Variables	Participants	BRI^a^			*P* value
		Tertile 1 (<3.87; n=4599)	Tertile 2 (approximately 3.87-5.61; n=4599)	Tertile 3 (≥5.61; n=4600)	
Age (y), mean (SD)	39.62 (11.43)	35.57 (11.38)	41.03 (10.89)	42.51 (10.79)	<.001
Sex, n (%)					<.001
Female	6761 (49.01)	2104 (49.37)	1975 (42.72)	2682 (55.38)	
Male	7037 (50.99)	2495 (50.63)	2624 (57.28)	1918 (44.62)	
PIR^b^, mean (SD)	3.019 (1.65)	3.096 (1.67)	3.130 (1.64)	2.817 (1.63)	<.001
Race or ethnicity, n (%)					<.001
Mexican American	2252 (8.97)	434 (5.30)	910 (10.53)	908 (11.28)	
Non-Hispanic White	6214 (67.83)	2243 (70.50)	2029 (68.39)	1942 (64.30)	
Non-Hispanic Black	2964 (11.35)	973 (10.73)	869 (9.65)	1122 (13.84)	
Other races or ethnicities	2368 (11.86)	949 (13.47)	791 (11.42)	628 (10.58)	
Educational level, n (%)					<.001
College graduate or higher	3383 (29.59)	1461 (36.93)	1138 (30.30)	784 (20.85)	
High school or less	5953 (36.96)	1661 (30.77)	1985 (36.56)	2307 (44.11)	
Some college	4462 (33.45)	1477 (32.31)	1476 (33.14)	1509 (35.03)	
Marital status, n (%)					<.001
Living with partner	1367 (9.07)	508 (10.57)	473(8.92)	386 (7.59)	
Married	6931 (54.72)	1964 (47.32)	2584 (60.30)	2383 (56.78)	
Never married	3420 (22.16)	1594 (31.26)	863 (16.73)	963(18.09)	
Other	2080 (14.06)	533 (10.85)	679 (14.05)	868 (17.54)	
Waist circumference (cm), mean (SD)	97.379 (16.41)	81.803 (7.16)	96.541 (6.90)	115.227 (12.70)	<.001
BMI (kg/m^2^), mean (SD)	28.633 (6.74)	22.662 (2.49)	27.781 (2.56)	36.044 (5.94)	<.001
Psoriasis, n (%)					<.001
No	13,429 (96.98)	4510 (97.88)	4471 (96.73)	4448 (96.28)	
Yes	369 (3.02)	89 (2.12)	128 (3.27)	152 (3.72)	
CVD^c^, n (%)					<.001
No	13,215 (96.17)	4502(98.16)	4434 (96.55)	4279 (93.58)	
Yes	583 (3.83)	97 (1.84)	165 (3.45)	321 (6.42)	
Metabolic syndrome, n (%)					<.001
No	10,440 (75.86)	4523 (98.50)	3695 (79.94)	2222 (46.83)	
Yes	3358 (24.14)	76 (1.50)	904 (20.06)	2378 (53.17)	
Diabetes (%)					<.001
No	11,648 (86.40)	4357 (95.68)	3976 (88.45)	3315 (74.10)	
Yes	1346 (8.05)	116 (2.07)	320 (5.09)	910 (17.75)	
Smoke status (%)					<.001
Former	2418 (19.57)	647 (16.10)	894 (21.27)	877 (21.53)	
Never	7760 (55.13)	2608 (56.07)	2555 (54.24)	2597 (55.08)	
Current	3620 (25.29)	1344 (27.83)	1150 (24.49)	1126 (23.39)	
Alcohol drinking status, n (%)					<.001
Former	1838 (12.31)	424 (8.05)	597 (11.88)	817 (17.41)	
Heavy	3695 (26.55)	1279 (27.85)	1255 (27.19)	1161 (24.45)	
Light	4201 (32.71)	1510 (34.12)	1462 (34.61)	1229 (29.15)	
Moderate	2478 (19.19)	910 (21.49)	818 (18.36)	750 (17.58)	
Never	1586 (9.23)	476 (8.49)	467 (7.96)	643 (11.41)	

a BRI: body roundness index.

b PIR: family poverty income ratio.

c CVD: cardiovascular disease.

BMI and WC levels increased significantly with higher BRI levels (*P*<.001). Regarding health status, the prevalence of psoriasis also rose, with 152 of 4600 (3.7%) participants in tertile 3 compared to 89 of 4599 (2.1%) in tertile 1 (*P*<.001). In addition, comorbidities were more frequent in tertile 3, including CVD in 321 of 4600 (6.4%) participants, metabolic syndrome in 2378 (53.2%), and diabetes in 910 (17.8%; all *P*<.001).

In terms of lifestyle factors, participants in tertile 3 were more likely to report never smoking (2597/4600, 55.1%) and light alcohol consumption (1229/4600, 29.2%) compared with tertile 1 (lowest BRI*; P*<.001).

### Association Between BRI and Risk of Psoriasis

As shown in Table 2, the BRI was distinctly linked to an increased risk of psoriasis across all models. Model 1 (unadjusted): each 1-unit increase in BRI was associated with a 10% increase in the odds of psoriasis (OR 1.10, 95% CI 1.05‐1.14; *P*<.001). Model 2 (adjusted for demographic variables such as age, sex, race or ethnicity, education level, PIR, and marital status): the association slightly attenuated but remained statistically significant (OR 1.09, 95% CI 1.04‐1.14; *P*<.001). Model 3 (fully adjusted model: further controlling for NHANES survey cycle, metabolic syndrome, diabetes, CVD, smoking status, and alcohol consumption): the positive association remained robust and significant, with each unit increase in BRI corresponding to an 11% increased risk of psoriasis (OR 1.11, 95% CI 1.05‐1.17; *P*<.001).

When BRI was divided into tertiles, the risk of psoriasis increased with BRI levels (*P*=.04). Compared to the lowest BRI tertile (<3.87, as the reference group), the second tertile (3.87≤BRI<5.613) showed linkage with a significantly higher risk of psoriasis (model 3: OR 1.49, 95% CI 1.03‐2.15*; P*=.04). The highest BRI tertile (≥5.61) showed an even greater increase in risk of psoriasis (model 3: OR 1.73, 95% CI 1.22‐2.45; *P*=.003; Table 2).

**Table 2.** Associations of the body roundness index (BRI) with psoriasis among participants in the National Health and Nutrition Examination Survey (NHANES) 2003‐2006 and 2009‐2014 cycles.

	Model 1^a^		Model 2^b^		Model 3^c^	
	OR^d^ (95% CI)	*P* value	OR (95% CI)	*P*-value	OR (95% CI)	*P* value
Per 1 unit increase	1.1 (1.05-1.14)	<.001	1.09 (1.04-1.14)	<.001	1.11 (1.05-1.17)	<.001
Tertile						
Tertile 1 (BRI:<3.87)	Reference		Reference		Reference	
Tertile 2 (BRI:3.87 to<5.61)	1.56 (1.10-2.20)	01	1.50 (1.05-2.14)	.03	1.49 (1.03-2.15)	.04
Tertile 3 (BRI:≥5.61)	1.78 (1.3-2.43)	<.001	1.72 (1.23-2.42)	.002	1.73 (1.22-2.45)	.003
*P* for trend	—^e^	<.001	—	.002	—	.003

a Model 1: crude model.

b Model 2: adjusted for sociodemographic variables (age, sex, race or ethnicity, educational level, poverty-income ratio [PIR], and marital status).

c Model 3: adjusted for sociodemographic variables (age, sex, race or ethnicity, educational level, PIR, and marital status), NHANES cycles, metabolic syndrome, diabetes, cardiovascular disease (CVD), smoke and alcohol consumption.

d OR: odds ratio.

e not applicable.

**Figure 2.**
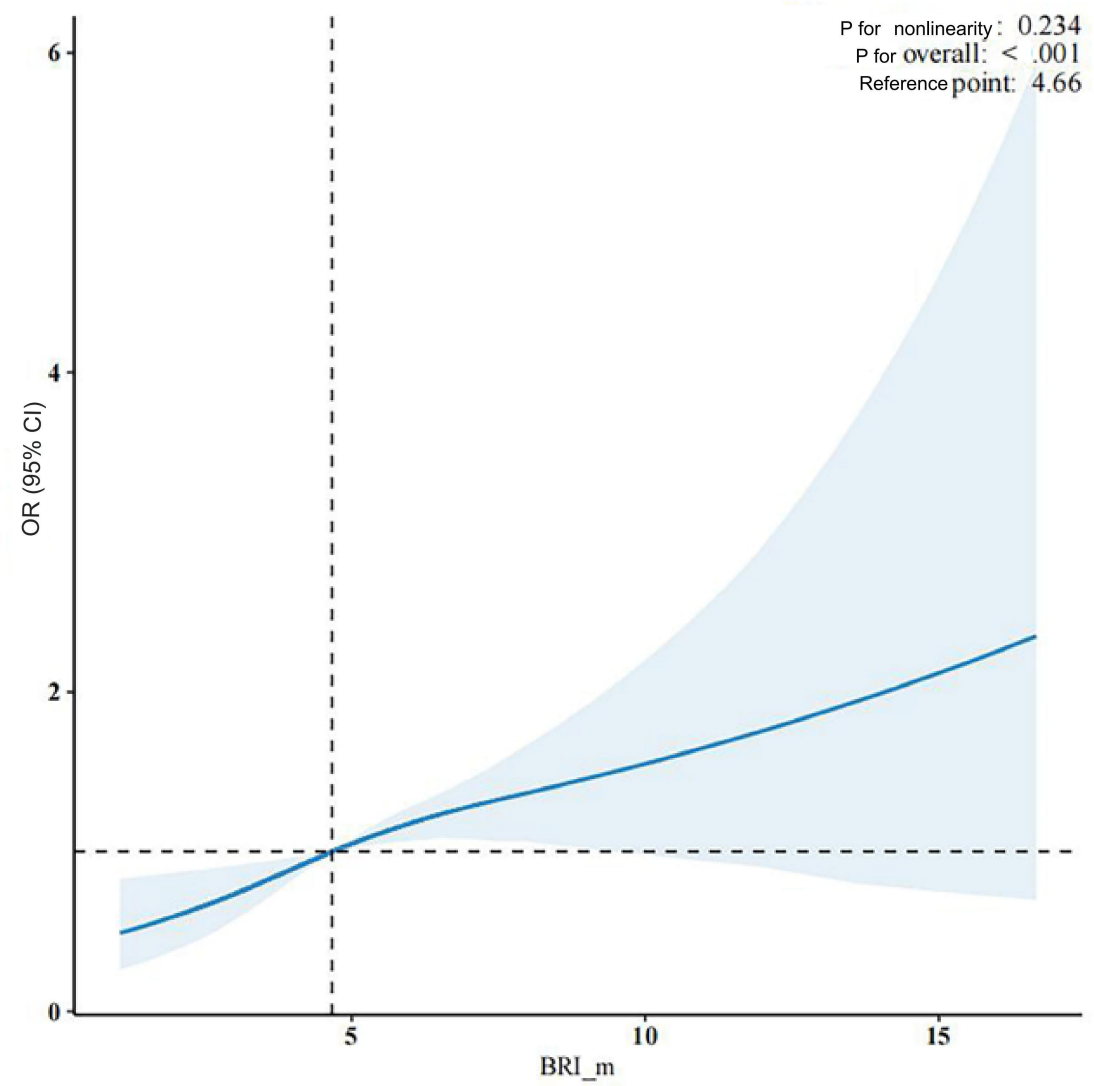
Weighted restricted cubic spline (RCS) analysis of the association between the body roundness index (BRI) and the risk of psoriasis. OR: odds ratio.

RCS analysis (Figure 2) demonstrated a significant linear association between BRI and risk of psoriasis, with no evidence of nonlinearity (*P* for nonlinearity=.23). Furthermore, this study conducted an in-depth analysis of the association between BFP and the risk of psoriasis. The results validated a notable positive correlation between BFP and risk of psoriasis, independent of confounding factors. In the unadjusted model, each 1% increase in BFP showed linkage with a 6% increase in risk of psoriasis (OR 1.06, 95% CI 1.04‐1.08; *P*<.001). After adjusting for demographic variables, the odds ratio decreased slightly to 1.05 (95% CI 1.03‐1.07; *P*<.001), and in the fully adjusted model—which included metabolic syndrome, diabetes, and other covariates—the association remained significant (OR 1.04, 95% CI 1.02‐1.06; *P*<.001). When analyzed in conjunction with BRI tertiles, participants in the middle and highest BRI tertiles had a 38% (OR 1.38, 95% CI 1.15‐1.66; *P*<.001) and 82% (OR 1.82, 95% CI 1.43‐2.32; *P*<.001) increased risk of psoriasis, respectively, relative to those in tertile 1 (lowest BRI)—suggesting that BRI may outperform BFP in predicting risk of psoriasis. Subgroup analyses further revealed that the association between BRI and psoriasis was stronger among individuals over the age of 40 years (OR 1.07, 95% CI 1.05‐1.09*; P*<.001). The risk was slightly higher in men (OR_male=1.06) than in women (OR_female=1.04; Table 3).

**Table 3.** Logistic regression analysis of body fat percentage (BFP) and psoriasis risk.

Variables		OR^a^ (95% CI)		*P* value
Model				
Model 1^b^ (BFP)		1.06 (1.04‐1.08)		<.001
Model 2^c^ (BFP)		1.05 (1.03‐1.07)		<.001
Model 3^d^ (BFP)		1.04 (1.02‐1.06)		<.001
Tertile				
Tertile 1^e^ (BRI^f^ :<3.87)		1.00		—^m^
Tertile 2 (BRI:3.87 to <5.61)		1.38 (1.15‐1.66)		<.001
Tertile 3 (BRI: ≥5.61)		1.82 (1.43‐2.32)		<.001
Age (y)				
<40		1.03 (1.01‐1.05)		<.01
≥40		1.07 (1.05‐1.09)		<.001
Sex				
Male		1.06 (1.04‐1.08)		<.001
Female		1.04 (1.02‐1.06)		<.001

a OR: odds ratio.

b Model 1: crude model.

c Model 2: adjusted for sociodemographic variables (age, sex, race or ethnicity, educational level, family income, and marital status).

d Model 3: adjusted for sociodemographic variables (age, sex, race or ethnicity, educational level, poverty-income ratio, and marital status), National Health and Nutrition Examination Survey (NHANES) cycles, metabolic syndrome, diabetes, cardiovascular disease, and smoke and alcohol consumption.

e Tertile 1 (BRI < 3.87) was used as the reference category; therefore, no 95% CI or *P* value was estimated.

f BRI: body roundness index.

g —: Tertile 1 was used as the reference group in all models; therefore, no odds ratio or 95% confidence interval was estimated for this category

### Association Between BRI and Risk of Psoriasis Across Subgroups

**Figure 3.**
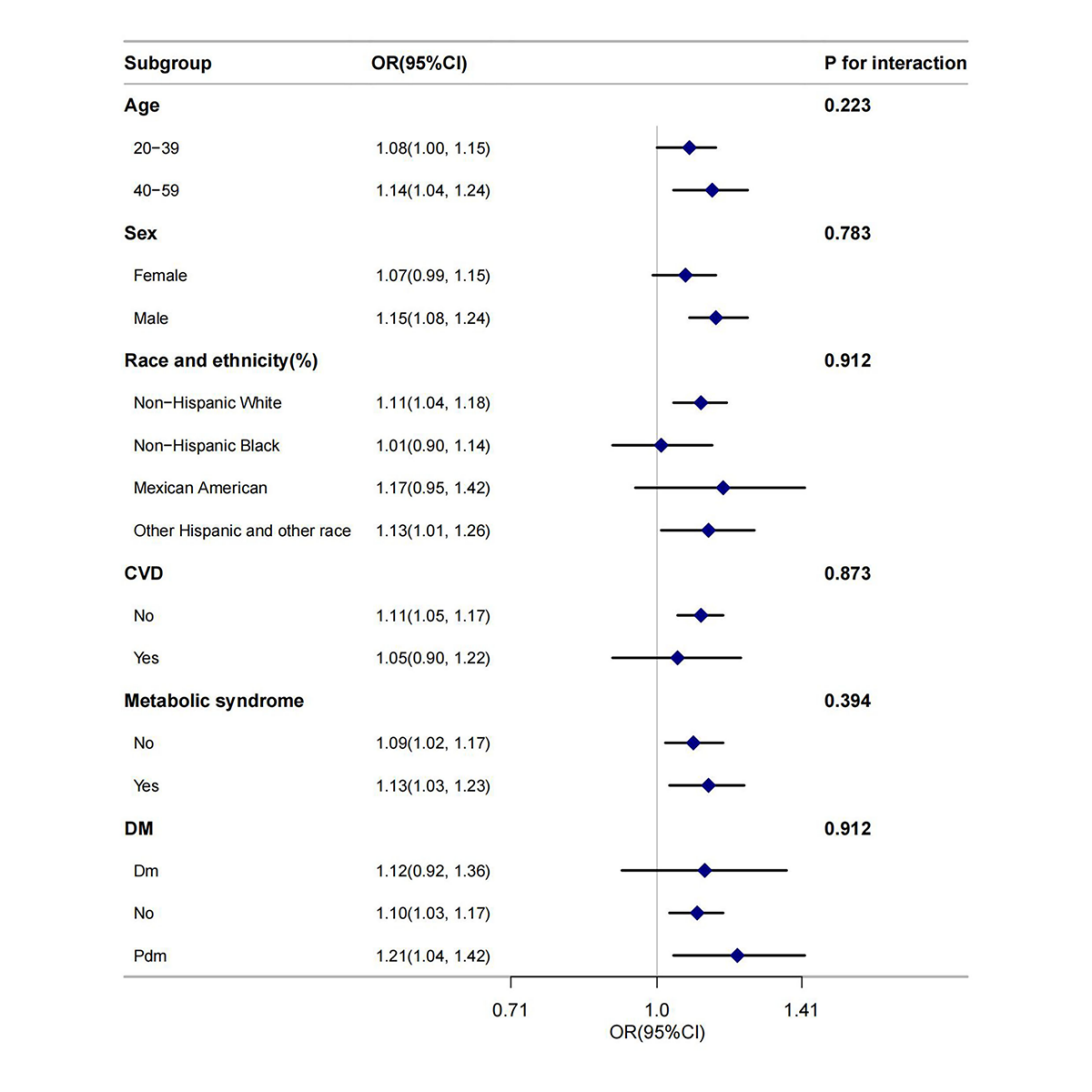
Forest plot analysis of the association between the body roundness index (BRI) and risk of psoriasis across different subgroups. CVD: cardiovascular disease; DM: diabetes mellitus; OR: odds ratio.

Subgroup analyses were implemented to assess the consistency of the association between BRI and risk of psoriasis across different population groups (Figure 3). Stratification was applied by age, sex, race or ethnicity, CVD, metabolic syndrome, and diabetes status, to explore potential effect modification. Across all subgroups, BRI remained significantly and positively associated with risk of psoriasis, with no statistically significant interactions noted (*P* for interaction >.05), indicating a stable association across population strata.

In the 40‐59 years age group, the OR was 1.14 (95% CI 1.04‐1.24), slightly higher than the OR of 1.08 (95% CI 1.00‐1.15) observed in the 20‐39 years age group (*P* for interaction=.22). For sex-based subgroups, the ORs were 1.15 (95% CI 1.08‐1.24) in males and 1.07 (95% CI 0.99‐1.15) in females (*P*=.78), indicating no significant sex-specific differences. Among participants with metabolic syndrome, the OR was 1.13 (95% CI 1.03‐1.23), compared to 1.09 (95% CI 1.02‐1.17) in those without metabolic syndrome (*P* for interaction=.39), suggesting consistent associations across metabolic health status.

### Comparison of BRI With Traditional Body Measurement Indicators (BMI and WC)

To further evaluate the predictive value of BRI and compare it with traditional anthropometric measures—BMI and WC—we conducted multivariable logistic regression using the fully adjusted model 3. For each 1-unit increase in BRI, the risk of psoriasis increased by 11% (OR 1.11, 95% CI 1.05‐1.17; *P*<.001). In comparison, each 1-unit increase in BMI was associated with a 3% increased risk (OR 1.03, 95% CI 1.02‐1.05; *P*<.001). Each 1-unit increase in WC conferred only a 1% increased risk (OR 1.01, 95% CI 1.01‐1.02; *P*<.001; Table 4).

**Figure 4.**
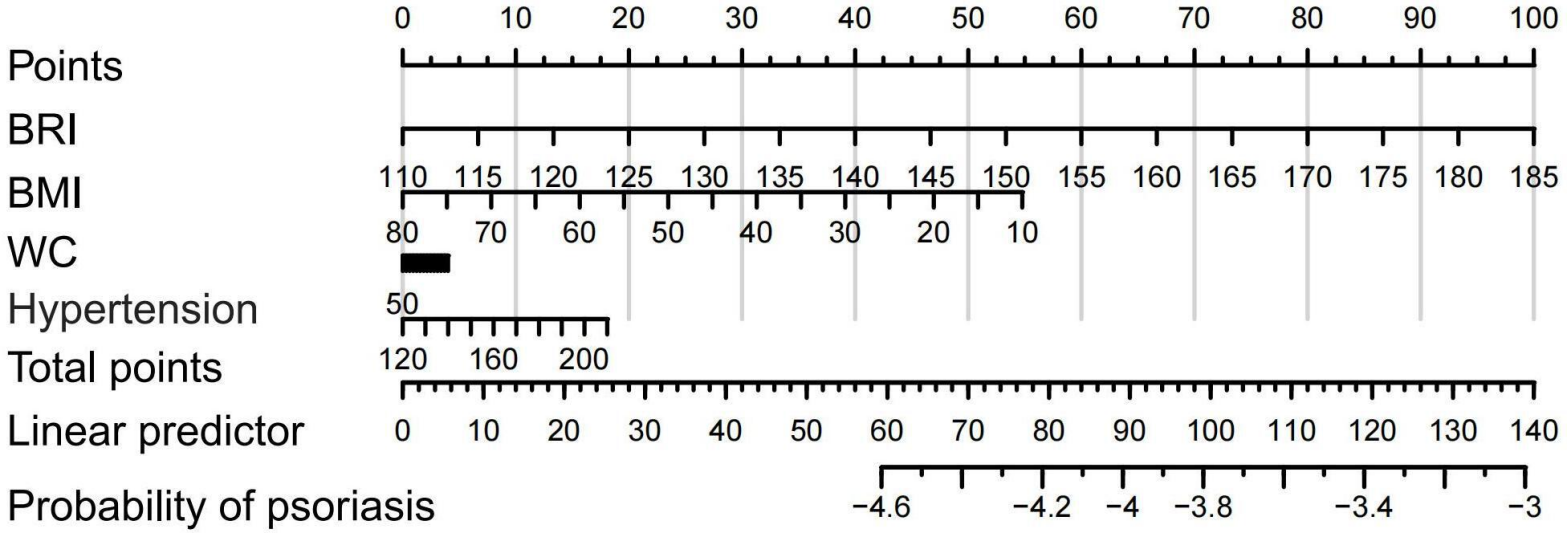
Nomogram constructed based on body roundness index (BRI), BMI, and waist circumference (WC).

To visualize and quantify the relative contributions of these indicators, we constructed a nomogram model based on multivariable logistic regression. The nomogram illustrated that the BRI score axis had the widest range, indicating that BRI contributed the most to risk of psoriasis prediction. In contrast, BMI and WC had shorter score axes, reflecting their smaller relative contributions (Figure 4).

**Table 4.** Logistic regression analysis.

Variables	Odds ratio (95% CI)	*P* value
Body roundness index	1.11 (1.05‐1.17)	<.001
BMI	1.03 (1.02‐1.05)	<.001
Waist circumference	1.01 (1.01‐1.02)	<.001

## Discussion

### Principal Findings

Psoriasis is a long-standing inflammatory skin condition that has been closely linked to metabolic disorders such as obesity, diabetes, and CVD [1,2,5,6]. Prior research has demonstrated significant links between risk of psoriasis and a range of anthropometric indicators such as BMI, WC, waist-to-hip ratio (WHR), and BRI [25,40]. However, most of this research has focused on individual obesity indicators in isolation, lacking a comprehensive comparison of their predictive value. Furthermore, many analyses did not sufficiently adjust for confounding factors that could influence observed associations. Using large-scale data from the NHANES, this study is the first to systematically compare the predictive performance of BRI, BMI, and WC in relation to the risk of psoriasis. The results suggest that BRI demonstrates higher sensitivity and greater clinical use than the other indices examined.

Prior research has consistently reported a positive correlation between BMI and risk of psoriasis. Obesity is thought to influence both the onset and severity of psoriasis through mechanisms such as chronic low-grade inflammation, elevated levels of proinflammatory cytokines, insulin resistance, and broader metabolic disturbances [41,42]. Despite its widespread use, BMI reflects only overall body mass without distinguishing between fat distribution types—particularly visceral versus subcutaneous fat—potentially limiting its ability to capture obesity-related metabolic risk [25]. Although WC is a better indicator of abdominal fat accumulation, it does not adjust for individual height differences. In contrast, BRI integrates both WC and height, enabling a more accurate estimation of total body fat distribution and metabolic burden [43]. Some studies have also suggested a J-shaped or U-shaped association between BMI and risk of psoriasis [44]. Our study used weighted RCS analysis and identified a stable linear association between BRI and risk of psoriasis (*P*=.23), reinforcing the potential of BRI as a reliable risk predictor. These findings are consistent with recent evidence [25].

The BRI, as an integrated measure of WC and height, is not only influenced by genetic and metabolic factors but also modifiable lifestyle factors, such as smoking, alcohol consumption, and physical activity [22]. In this analysis, participants in the highest BRI tertile were more frequently identified as light drinkers and never smokers, suggesting behavioral patterns may modulate body shape and fat distribution. Notably, prior studies have documented that smoking is linked to an increased visceral adiposity despite lower overall BMI, potentially elevating BRI scores through central fat accumulation [45]. Similarly, moderate to heavy alcohol consumption has been linked to higher WC and increased abdominal fat [46], which directly contributes to elevated BRI. Moreover, few studies have examined the distribution patterns of BRI across different population subgroups or its association with psoriasis. In this study, BRI demonstrated more stable trends than BMI and WC across demographic subgroups defined by age, sex, educational level, and marital status. Importantly, BRI showed a consistent positive association with psoriasis risk across all subgroups. Previous research has shown that higher BMI and WC are more prevalent among women, individuals with lower socioeconomic status, and those with limited educational backgrounds [2]. Our findings indicate that the distribution trends of BRI in these populations mirror those of BMI and WC. However, BRI may be more sensitive in capturing obesity-related metabolic risk [25]. Unlike prior studies that have largely focused on BMI and WC, this study uses NHANES data to systematically evaluate BRI distribution across diverse populations and its association with both psoriasis and metabolic disorders. These findings not only provide new insights into the complex interplay between obesity and inflammatory diseases such as psoriasis, but also underscore the significance of BRI as a practical clinical tool for metabolic health surveillance. In subgroup analyses, the association between BRI and psoriasis remained significant across various strata, including age, sex, race or ethnicity, diabetes, CVD, and metabolic syndrome. Education level, smoking status, and alcohol consumption were not included in the subgroup analyses due to potential multicollinearity with socioeconomic and lifestyle factors, as well as concerns about statistical power related to over-stratification. Although WHR and waist-to-height ratio (WHtR) are also widely adopted indicators of central obesity, they were not included in our analysis due to data limitations and a desire to minimize multicollinearity among closely related anthropometric indices. Moreover, BRI already incorporates WC and height into its calculation, thereby partially accounting for the components used in WHtR. Future studies could explore the predictive value of WHR and WHtR in combination with BRI to further refine obesity-related risk assessment models. In this analysis, lifestyle behaviors—including smoking and alcohol use—showed significant associations with BRI. Participants in the highest BRI tertile were more frequently identified as light alcohol consumers and individuals who had never smoked. These findings suggest that lifestyle factors may influence body shape and fat distribution, thereby indirectly impacting BRI values. This underscores the need to consider behavioral determinants when interpreting BRI in clinical and epidemiological contexts.

Although this study provides compelling evidence for the link between BRI and risk of psoriasis, the underlying biological mechanisms remain to be fully clarified. Prior research has suggested that obesity contributes to the pathogenesis of psoriasis through mechanisms such as chronic low-grade inflammation, elevated levels of proinflammatory cytokines, insulin resistance, and broader metabolic dysregulation [47-49]. As a novel anthropometric index, BRI may more accurately capture fat distribution, metabolic abnormalities, and systemic inflammatory burden relative to traditional measures such as BMI or WC, potentially making it more relevant to the pathophysiology of psoriasis. In this study, BRI remained a notable independent predictor of risk of psoriasis even after adjusting for multiple confounding factors, including metabolic syndrome, diabetes, and CVD. This reinforces its potential clinical value in identifying individuals at elevated risk for inflammation-related conditions. Future research should aim to elucidate the biological pathways underlying this association by integrating multiomics approaches—such as metabolomics, inflammatory biomarker profiling, and gut microbiome analysis—which may offer deeper insight into the mechanistic links between BRI and psoriasis.

This study highlights a strong and consistent association between elevated BRI and an increased likelihood of psoriasis. This association remained stable under various model adjustments and statistical frameworks, suggesting it is not merely attributable to confounding influences. Importantly, the risk of psoriasis rose progressively with increasing BRI, indicating a clear linear trend. These observations support the potential of BRI as a meaningful indicator for identifying individuals with heightened susceptibility to psoriasis. Given its capacity to reflect both fat distribution and metabolic status, BRI may serve as a useful addition to existing risk assessment tools. Future studies should delve deeper into the mechanistic pathways linking BRI and psoriatic disease and evaluate its relevance in clinical prediction models and preventive strategies.

Despite offering compelling evidence for the use of BRI in assessing psoriasis risk, this study contains several limitations. First, as it is based on cross-sectional data from NHANES, causality cannot be inferred; thus, longitudinal cohort studies are necessary to confirm the long-term impact of BRI on psoriasis development and progression. Second, while BRI is a useful surrogate for fat distribution, it does not directly measure visceral adiposity or the muscle-to-fat ratio. Future research incorporating advanced imaging modalities such as dual-energy X-ray absorptiometry or magnetic resonance imaging could yield more precise assessments of body composition and further clarify the biological relevance of BRI in the context of psoriasis. Finally, as this study is based on US NHANES data, the findings may not be directly generalizable to populations in other countries with different ethnic, dietary, and health care backgrounds. Although subgroup analysis within the US population revealed consistent associations across various demographic groups, future studies should validate these results using international datasets. Cross-country comparisons could help evaluate the universality of BRI as a biomarker for psoriasis risk.

### Conclusions

Using large-scale NHANES population data, this study systematically evaluated the association between the BRI and psoriasis risk, and, for the first time, directly compared the predictive performance of BRI, BMI, and WC within a unified analytical framework. The results indicate that BRI may offer greater sensitivity in predicting psoriasis risk. Unlike traditional anthropometric measures, BRI integrates both height and WC, providing a more accurate representation of fat distribution and metabolic burden. Furthermore, weighted RCS analysis confirmed a stable linear association between BRI and psoriasis risk, and BRI consistently showed strong predictive value across diverse demographic subgroups. These findings highlight the potential clinical use of BRI as an early risk assessment tool for psoriasis.

**Figure 5.**
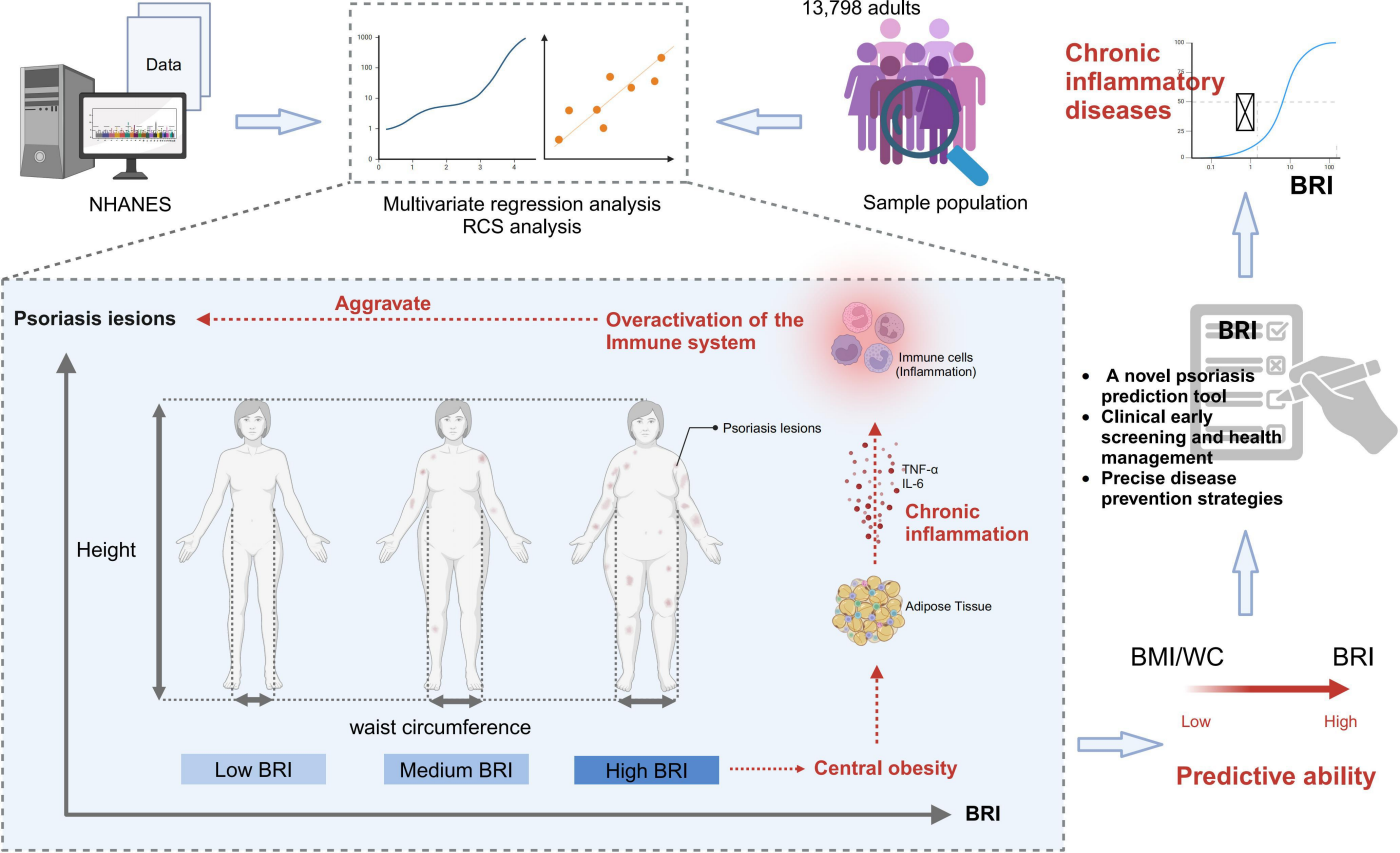
Conceptual framework illustrating the association between body roundness index (BRI) and psoriasis risk. BRI: body roundness index; NHANES: National Health and Nutrition Examination Survey; RCS: restricted cubic spline; WC: waist circumference.

To advance this line of research, future investigations should delve into unraveling the biological pathways linking BRI to psoriatic pathophysiology—particularly in the context of chronic inflammation, metabolic dysregulation, and fat distribution. Given the cross-sectional nature here, prospective cohort studies are essential to validate the predictive value of BRI for psoriasis onset and progression across different populations. In addition, integrating multiomics data—including metabolomics, inflammatory markers, and microbiome profiles—may further enhance the use of BRI in personalized health monitoring and disease prevention, offering new strategies for early diagnosis and targeted intervention in psoriasis (Figure 5).

## Supplementary material

10.2196/75727 Multimedia Appendix 1 Diagram illustrating the measurement method and grouping of body roundness index (BRI).

## Abbreviations

BFP: body fat percentage
BRI: body roundness index
CVD: cardiovascular disease
MEC: mobile examination center
NCHS: National Center for Health Statistics
NHANES: National Health and Nutrition Examination Survey
OR: odds ratio
PIR: poverty-income ratio
RCS: restricted cubic spline
STROBE: Strengthening the Reporting of Observational Studies in Epidemiology
WC: waist circumference
WHR: waist-to-hip ratio
WHR: waist-to-hip ratio
WHtR: waist-to-height ratio
