# Supplementary figures and images for: Evaluating the Body Roundness Index as a Novel Digital Biomarker for Psoriasis Risk Prediction: Cross-Sectional Study
Source: JMIR Med Inform. 2025 Dec 23;13:e75727. doi: 10.2196/75727 (PMC12724066; doi:10.2196/75727)

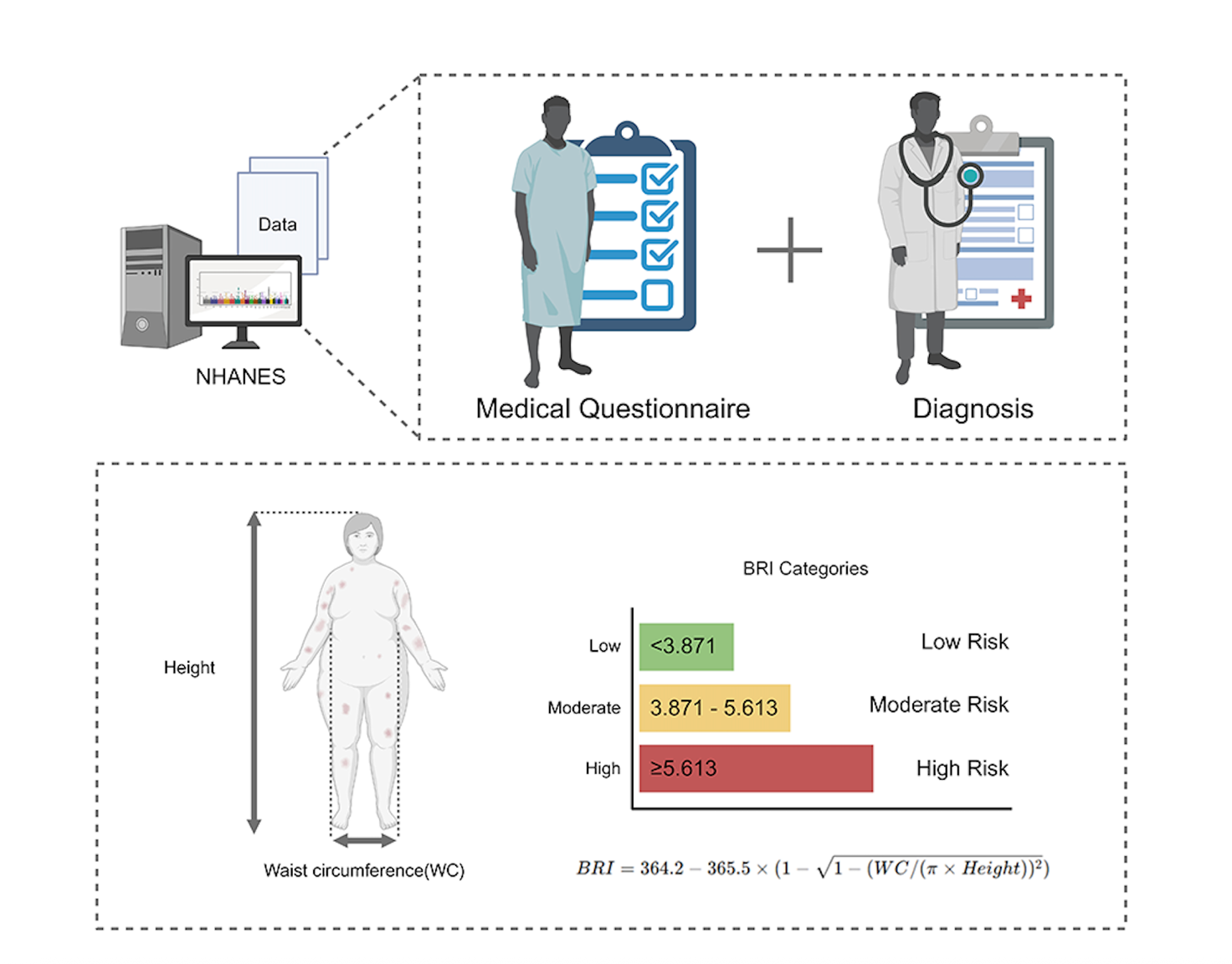


Figure S1 Diagram illustrating the measurement method and grouping of BRI.

Supplement: Multimedia Appendix 1 [file medinform-v13-e75727-s001.docx]
